# Design and Analysis of a Sensor System for Cutting Force Measurement in Machining Processes

**DOI:** 10.3390/s16010070

**Published:** 2016-01-07

**Authors:** Qiaokang Liang, Dan Zhang, Gianmarc Coppola, Jianxu Mao, Wei Sun, Yaonan Wang, Yunjian Ge

**Affiliations:** 1 College of Electric and Information Technology, Hunan University, Changsha 410082, Hunan, China; mao_jianxu@126.com; 2 State Key Laboratory of Advanced Design and Manufacturing for Vehicle Body, Hunan University, Changsha 410082, Hunan, China; 3 Lassonde School of Engineering, York University, Toronto, ON M3J 1P3, Canada; dan.zhang@lassonde.yorku.ca (D.Z.); Gianmarc.Coppola@uoit.ca (G.C.); 4 Institute of Intelligent Machines, Chinese Academy of Science, Hefei 230031, Anhui, China; yjge@iim.ac.cn

**Keywords:** cutting force measurement, multi-component, in-process measurement, sensor system

## Abstract

Multi-component force sensors have infiltrated a wide variety of automation products since the 1970s. However, one seldom finds full-component sensor systems available in the market for cutting force measurement in machine processes. In this paper, a new six-component sensor system with a compact monolithic elastic element (EE) is designed and developed to detect the tangential cutting forces F*x*, F*y* and F*z* (*i.e.*, forces along *x*-, *y*-, and *z*-axis) as well as the cutting moments M*x*, M*y* and M*z* (*i.e.*, moments about *x*-, *y*-, and *z*-axis) simultaneously. Optimal structural parameters of the EE are carefully designed via simulation-driven optimization. Moreover, a prototype sensor system is fabricated, which is applied to a 5-axis parallel kinematic machining center. Calibration experimental results demonstrate that the system is capable of measuring cutting forces and moments with good linearity while minimizing coupling error. Both the Finite Element Analysis (FEA) and calibration experimental studies validate the high performance of the proposed sensor system that is expected to be adopted into machining processes.

## 1. Introduction

Computer Numerical Control (CNC) machines are operated by precisely programmed commands and widely used in modern manufacturing industry, enabling the manufacture of complex-shaped products or parts that cannot be produced by manually operated machines.

Nowadays, every manufacturer in the global market is pitted against worldwide competitors with consistently improving product quality, enhanced manufacturing productivity, elimination of inspections, and shrinking total machining costs.

The condition of cutting tools and the cutting process should be identified without human assistance and/or interrupting the manufacturing process operation, which is one of the most important operating criteria that influences the manufacturing quality and productivity. An automated machining process with a successful machining condition monitoring system could allow increased principal equipment utilization, hence achieving considerable cost savings for the manufacturing industry [1,2].

As precise estimation and determination of cutting force at tool tips in manufacturing processes is the most effective method for monitoring machine tools and the machining processes, it is indispensable in many research and application fields for monitoring the tool conditions, analyzing of machining methods and tools, estimating real-time tool wear [3], designing proper machining tools, characterizing and optimizing manufacturing process and cutting parameters, *etc.* Consequently, sensor systems for measuring cutting force are receiving widespread attention within the equipment manufacturing and other related industries community.

The cutting forces during machining process are dependent on many parameters such as depth of cut, configuration of the cutting tool, feed rate, material of the workpiece and the tool, and some unknown factors such as cooling method. Therefore, theoretical cutting force calculations are hardly applicable to monitor practical machining processes in a factory environment.

A great number of sensor systems with many sophisticated signal and information processing techniques proposed for the pertinent applications can be found in the literature. Additionally, a lot of commercial devices capable of measuring the cutting force during milling, turning, drilling or grinding have been developed by many companies such as Kistler TLC (Technische Lehrmittel Construktion, Unna, Germany), *etc*.

Conveniently, the cutting force measurement systems could be separated into at least two groups, consisting of indirect and direct approaches. The direct measurement approach refers to detecting and quantifying the cutting force by using force dynamometers directly. This is done by relying on some specific effects such as mechano-magnetic, mechano-electric, and mechano-optic conversions, while the indirect measuring approach refers to a cutting force that is estimated by dynamic parameters associated with cutting force such as acceleration, acoustic emission, vibration, and spindle motor current. The most common indirect approaches to cutting force measurement or monitoring are based upon motor current and power measurement [4,5]. Shin *et al.* [6] designed an indirect approach for measuring cutting force in end-milling process with a three-axis acceleration sensor and Hall-effect current sensors. An indirect approach for detecting cutting force for an Adaptive Control with Constraints (ACC) system for machining centers by using the currents of a.c. feed-drive servo drives was reported in [7]. A novel approach for measuring cutting force via command voltages drawn by electro-magnetic bearing was proposed by Auchet *et al.* [8], and the experimental results show that the calculated cutting forces are in good agreement with commercial Kistler measurement system. The static accuracy and bandwidth of the Altintas *et al.* presented a comprehensive model that evaluates the cutting force in normal and tangential directions through modeling the cutting mechanics, feed variation and tool indentation effect [9]. In recent years, different methods for evaluating or predicting cutting forces based on tool indentation effect [10], nonlinear cutting force coefficients [11], and rotational DOF (Degrees of Freedom) of the multi-axis kinematics effect on mechanics of the cutting process [12] are considerably attracting the attentions of researchers. Within most environments the indirect approach is easier to achieve, but has several limitations such as high computation time, unsuitable for multi-axis cutting process, does not consider the frictional behavior of the machine tools.

On the other hand, direct measuring approaches have been widely employed because they can provide more accurate measurement of cutting forces. Although, they have their limitations like high cost, layout constraints, fragility to overload, *etc.* Rao, Gao, and Friedrich presented an integrated force measurement method for on-line cutting geometry inspection, and a piezoelectric force sensor with high resolution (0.44 mN) and sensitivity (7 mV/gm) which was adopted to measure the feed force combined with thrusts during the diamond-cutting process [13]. A novel cylindrical capacitive displacement sensor and a magnetic exciter for monitoring end milling processes were proposed by Kim *et al.* [14]. It was verified by cutting experiments that the proposed sensory system was suitable in the monitoring of high speed cutting conditions on various machine tools. A strain gauge based tool dynamometer and a piezo-film accelerometer, with a natural frequency of 2.35 kHz, was developed to detect dynamic and static cutting force for ultra-precision machine tools [15]. Jin, Venuvinod, and Wang proposed an approach for detecting cutting force with an optical fiber sensor, and the results of calibration experiment and manufacturing tests show the proposed system obtains satisfactory performances (Its static sensitivity and linearity of the system are 2.51 mV/N and 1.2% Full Scale (F.S.), respectively, with a natural frequency of 950 Hz) [16]. The pros and cons as well as typical designs of the mentioned intrinsic transduction techniques are summarized in the Table 1. These transduction techniques are commonly interconnected in practical cutting forces measurement systems [17,18].

**Table 1:** Intrinsic transduction techniques for cutting force measurement.

Measuring Technology	Direct/Indirect Measurement	Pros	Cons	Typical Designs
Current	indirect	easier to achieve	time-consuming	[5]
		cost effective	unsuitable for multi-axis cutting process	
			without consideration of the frictional behavior of the machine tools	
Voltage	indirect	wide bandwidth (up to 4 kHz)	Limited to stable conditions	[8]
		easy to conversion and processing	susceptible to electromagnetic interference	
Strain gauge	direct	simple construction	higher power consumption	[15]
		high and adjustable resolution	rigid and fragile	
		high reliability	scarce reproducibility	
Capacitive	direct	high sensitivity and resolution	temperature sensitive	[14]
		long-time stability	stray capacitance	
		Adaptability to Environment	Edge effect	
Optoelectronic	direct	good reliability	non-conformable	[16]
		wide measurement range	hard to construct dense arrays	
		good adaptability to workshop conditions		
Piezoelectric	direct	high frequency response and high dynamic range rangeability higher accuracy and finer resolution high sensitivity and stiffness	charge leakages	[13]
			poor spatial resolution	
			deteriorations of voltages or drifts in the presence of static forces	

Many different sensory systems for cutting force measurement have been developed for specific tasks. However, many tasks such as monitoring and controlling of high speed machining processes require reliable and practical sensing methods to measure the cutting force, which cannot be achieved by the majority of the present sensory systems. This is partially due to limited workpiece sizes, low frequency bandwidths, mounting constraints, wiring complexities, and susceptibility to harsh machining environments [19]. In this paper, a novel six-component Force/Moment (F/M) sensor is designed to detect the normal and tangential cutting force (F*x*, F*y* and F*z*), as well as the cutting moments (M*x*, M*y* and M*z*), simultaneously.

## 2. System Configuration

**Figure 1:**
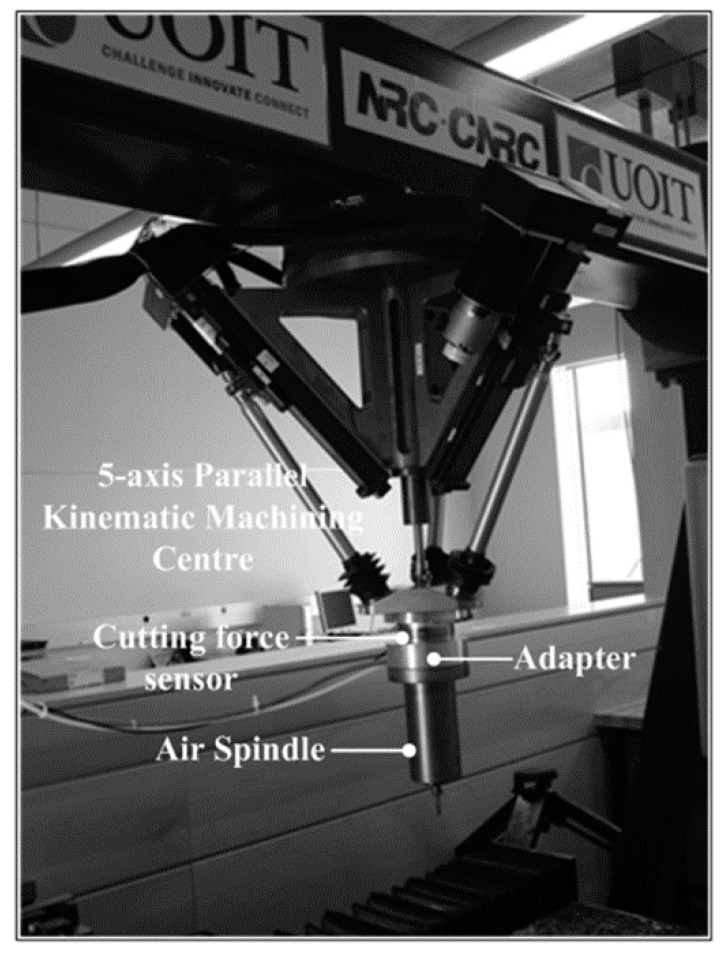
Experimental setup for implementing the sensor system for cutting force measurement.

The experimental setup for implementing the sensor system for cutting force measurement is shown in Figure 1. The six-component cutting F/M sensor is sandwiched between the adapter and the moving platform of a 5-axis parallel kinematic machining center. After calibration, the multi-component F/M sensor is capable of measuring the normal and tangential cutting force (F*x*, F*y* and F*z*), as well as the cutting moments (M*x*, M*y* and M*z*), simultaneously.

**Figure 2:**
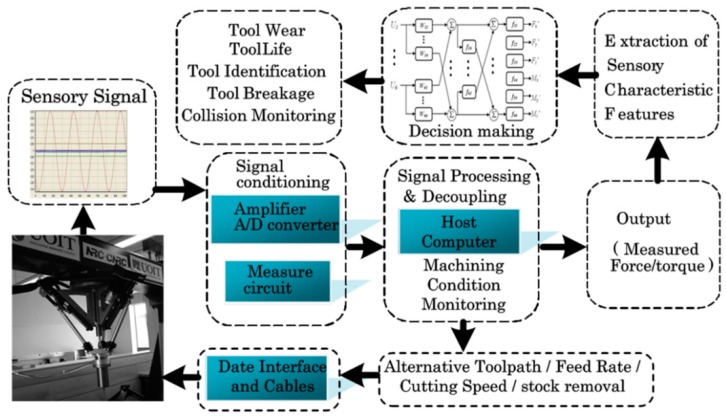
Schematic illustration of strategy of the system.

Raw signals from the sensor are processed, stored and transmitted to the host-computer by the integrated electrical circuit board as shown in Figure 2. Feature extraction of signals with the appropriate signal processing algorithms is adopted to obtain a simplified signal and specific characteristic features related to the cutter conditions. Decision-making strategies analyze the characteristic features and perform a pattern association task. Artificial neural networks, fuzzy logic system, expert systems, and other hybrid intelligent systems are proving their effectiveness in decision making of condition monitoring systems to make sure efficient removal of metals and taking correct action to prevent harm in the event of breakages or accidents. The input signals were also used to modify the part of the program that is used in the second pass with alternative tool path, feed rate, cutting speed, stock removal.

## 3. Design and Construction of the Sensor

The most common technique to detect cutting F/M relies on resistive measurement approach with strain gauges. This is due to its advantages such as high reliability with time, excellent linearity over wide range, simplicity, wide operating temperature range, adjustable resolution, maintenance free and relatively low in cost, *etc.* [20]. The sensing system based on resistive approach consists of four functional elements: strain gauges, Elastic element (EE) with a specially designed shape, measuring bridges, and electric circuit.

### 3.1. Sensing Principle

The strain gauges bonded onto the EE are sensitive to dimensional changes when a cutting force is applied to the sensor, and their resistances change according to:

(1) $$\frac{\Delta R_{i}}{R_{i}}=G\varepsilon_{i}$$

where Δ*R_i_*/*R_i_* is the variation of relative resistance ratio of the *i*th strain gauge. *G* and *ε_i_* are the gauge factor of the strain gauge and specific strain, respectively. Provided that the EE behaves within the elastic range of the material, the strains and the applied cutting load are related by the following equation:

(2) $$\varepsilon_{i}=f_{i}(F)$$

where $F\in {ℜ}^{6}$ is the cutting load vector that contains the forces **F***_s_* and the moments **M***_s_* applied onto the sensor.

Wheatstone Bridge arrangements are generally employed as measuring bridges via electrically connecting several strain gauges (generally 4, 8, or a still greater number) to measure the small resistive changes with high sensitivity and inherent linearity. Therefore, there is a relation between the output voltage variation of the measuring bridges and the strains due to the applied cutting load:

(3) $$\Delta V_{0}=V_{E}GW\varepsilon =TF$$

where $\Delta V_{0}\in {ℜ}^{6}$, *V_E_* is the voltage excitation source of the bridges, $W\in {ℜ}^{6\times 24}$ and $T\in {ℜ}^{6\times 6}$ represent the bridge transformation matrix and the transducer matrix, respectively. The transducer matrix is a constant matrix that depends on the configuration of the measuring bridges, the structure of the EE, and the specific location of the strain gauges on the EE. After calibration or structural analysis, the applied cutting load can be determined and calculated:

(4) $$F=T^{\#}\Delta V_{0}+(I-T^{\#}T)z$$

where $T^{\#}$ is the generalized inversion of **T**. **z** is an arbitrary 6 × 1 vector, and usually set as **z** = **o**.

Multi-component cutting F/M sensor should be designed to be equally sensitive or accurate among its all force and moment components. As a consequence, the sensor and its electrical circuit should be characterized with simple but effective decoupling methods, identical amplification among components associated with a considerable degree of integration. The transducer matrix is normalized to avoid the unit inconsistency problem:

(5) $$\bar{T}=N_{VN}^{-1}TN_{FN}$$

with normalization compensates:

(6) $${Ν}_{VN}=\mathrm{diag}\{\Delta V_{1M},\Delta V_{2M},\Delta V_{3M},\Delta V_{4M},\Delta V_{5M},\Delta V_{6M}\}$$

(7) $${Ν}_{FN}=\mathrm{diag}\{F_{xM},F_{yM},F_{zM},M_{xM},M_{yM},M_{zM}\}$$

where △*V_iM_* and *F_iM_* (or *M_iM_*) are maximal voltage variation of the *i*th measuring bridge and pre-specified maximal cutting forces (or moments). The anisotropy index of the sensor can be obtained by the condition number of $\bar{T}$ [21]:

(8) $$C_{o}(\bar{T})=\frac{\sigma_{\max}(\bar{T})}{\sigma_{\min}(\bar{T})}$$

where $\sigma_{\max}$and $\sigma_{\min}$ represent the largest and the smallest singular values of $\bar{T}$, respectively.

The absolute sensitivity of the sensor can be evaluated by [22]:

(9) $$S_{o}=\text{trace}({\bar{T}}^{T}\bar{T})=\sum_{i=1}^{6}\sigma_{i}^{2}$$

or:

(10) $$S_{o}=\det({\bar{T}}^{T}\bar{T})=\prod_{i=1}^{6}\sigma_{i}$$

The obtained cutting load is respect to the sensor’s coordinate frame {**S**}, and should be transformed to the tool’s coordinate frame {**T**} as:

(11) $$F_{t}=J_{t}F$$

where:

(12) $$J_{t}=\left[\begin{array}{cc} R_{t} & O\\ S(r_{t})R_{t} & R_{t} \end{array}\right]$$

Here, **r_t_** is the location vector of the frame {**S**} with respect to the frame {**T**}, **R_t_** is the is the orientation matrix of frame {**S**} relative to the frame {**T**}, **O** is the null matrix, and **S**(**r_t_**) represents skew-symmetric matrix operator of vector **r_t_**.

### 3.2. Structure of the Proposed Six-Component Cutting F/M Sensor

**Figure 3:**
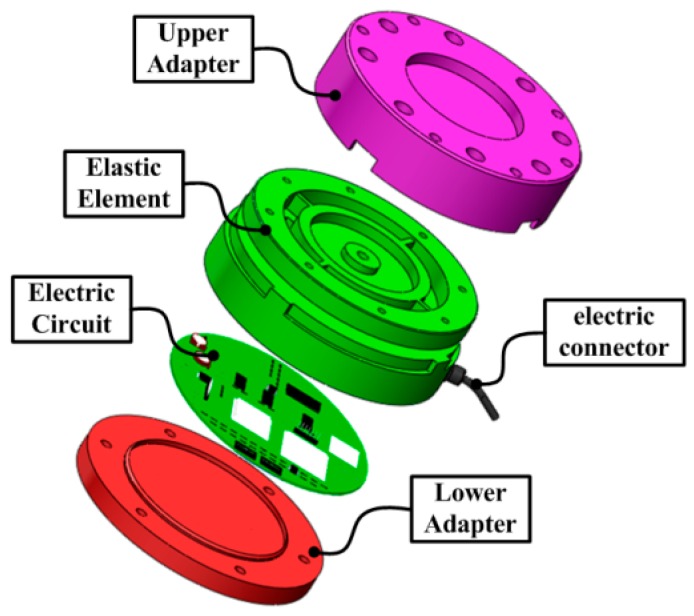
Structure of the proposed sensor.

The distinctive design of the proposed six-component cutting F/M sensor consists of four parts as illustrated in Figure 3. In particular, the upper and lower adapters are connected to the upper and lower portions of the EE by stainless steel screws with controlled torque, respectively. The integrated electric circuit is mounted in the EE, and electrically connected to host computer via the electric connector. The sensor is mounted to the spindle and the machine center via the upper and lower adapters, respectively.

### 3.3. Structure of Elastic Force-Sensing Elements

**Figure 4:**
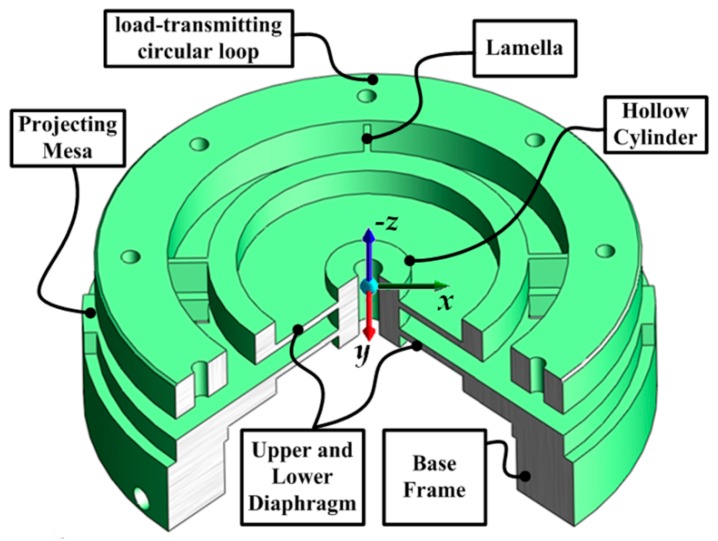
3D model of the monolithic EE structure.

Briefly, the monolithic EE consists of double circular diaphragms, connected by a center hollow cylinder, four lamellas whose axes are perpendicular to each other in a cross-shape, a load-transmitting circular loop that transmits the cutting load, and a base frame (as indicated in Figure 4). The base frame and the circular loop connect with the upper and lower adapters by bolts, respectively.

Practically, the EE transmits the applied cutting load into deformations of its flexible sensing portions through its double diaphragms and lamellas. In particular, the upper diaphragm that serves as an elastic portion is bonded with two groups of strain gauges, and it is responsive to the applied moment to be measured M*x*, M*y*. Similarly, the lower diaphragm that serves as an elastic portion is bonded with three groups of strain gauges, and it is responsive to the applied force F*x*, F*y*, and F*z*. Additionally, the lamellas that act as elastic portions are bonded with four strain gauges, and they are sensitive to the moment M*z*.

When the forces to be measured F*x*, F*y* or F*z* act on the sensor, the circular loop connected with the upper adapter floats, while the base frame conneted with the lower adapter acts as a fixed support. Consequently, corresponding elastic deformation and strain will take place on the lower diaphragm. Accordingly, when the moment to be measured M*x* (or M*y*) act on the sensor, elastic deformations mainly take place on the upper and lower circular diaphragms. When the moment to be measured, M*z*, acts on the load-transmitting circular loop, the inner borders of the lamellas provide fixed support while the outer borders of the lamellas float. Corresponding elastic deformation and strain consequently take place on the lamellas.

## 4. Optimal Determination of the Elastic Element Dimensions

### 4.1. Theoretical Analysis

The radial and tangential strains on the diaphragms induced by the applied cutting force can be expressed by:

(13) $$\xi_{r}(r)=-\frac{h}{2}\frac{d^{2}\zeta }{dr^{2}}=\frac{-3F(\varsigma +2)(1-\mu^{2})}{4\pi Eh^{2}}$$

(14) $$\xi_{t}(r)=-\frac{h}{2}\frac{d\zeta }{dr}=\frac{-3\varsigma F(1-\mu^{2})}{4\pi Eh^{2}}$$

where:

(15) $$\varsigma =2\ln\frac{2r}{d}-\iota -\frac{\iota d^{2}}{4r^{2}}$$

(16) $$\iota =\frac{4D^{2}}{d^{2}-D^{2}}\ln\frac{D}{2d}$$

where *E* and *µ* are the Young’s modulus and the Poisson’s ratio, respectively. *d*, *D*, and *h* are the inner circle diameter, the extended circle diameter, and the thickness of the diaphragm, respectively.

The corresponding radial and tangential stresses that occur on the diaphragm can be obtained by the expressions below:

(17) $$\vartheta_{r}(r)=\frac{E}{1-\mu^{2}}\left(\xi_{r}+\mu \xi_{t}\right)=\frac{-3F}{4\pi h^{2}}\left[(\mu +1)\varsigma +2\right]$$

(18) $$\vartheta_{t}(r)=\frac{E}{1-\mu^{2}}\left(\xi_{t}+\mu \xi_{r}\right)=\frac{-3F}{4\pi h^{2}}\left[(\mu +1)\varsigma +2\mu \right]$$

The radial strain and stress on the lamellas (of dimensions *l*, *b*, and *h*) induced by the applied cutting force can be expressed by:

(19) $$\xi_{r}(x)=-\frac{h}{2}\frac{d^{2}\zeta }{dx^{2}}=\frac{6F}{bh^{2}E}(\frac{3}{2}l-x)$$

(20) $$\xi_{t}(x)=-\frac{Eh}{2}\frac{d^{2}\zeta }{dx^{2}}=\frac{6F}{bh^{2}}(\frac{3}{2}l-x)$$

### 4.2. Simulation-Driven Optimization (SDO)

In general, multi-component force sensors always suffer from a critical drawback in the form of tradeoffs. These tradeoffs are typical in mechanical design and consist of a compromise between different performance characteristics such as sensitivity and stiffness, isotropy and coupling, static and dynamic performances. For example, optimum absolute sensor sensitivity corresponds to the desire of generating as much elastic strain as possible in the EE. However, both sensor sensitivity and general stiffness strongly depend on the geometrical parameters of the EE structure. More specifically, maximizing the sensor sensitivity is in contradiction with maximizing the general stiffness. Consequently, the optical design and development of a multi-component cutting F/M sensor is complicated and cannot be realized through direct optimization methods.

The SDO makes the procedure of new product development faster and more efficient. Design Exploration (DE) is a useful tool supplied by ANSYS Inc. (Pittsburgh, PA, USA), which could be performed to simulate the part response according to input parameter changes. Moreover, associated with response surface tools and optimization approaches, it can provide the most appropriate choices of input parameters to fulfill the system requirements. A design parameters study was performed via SDO to yield a set of feasible complete designs and make sure that the proposed sensor meets given performance requirements. A set of design parameters is defined by the independent geometric dimensions of the lamellas, the upper and lower diaphragms. Additionally, the max-deformation, the min-strain, the max-stress, and the primary response are set as output parameters. Table 2 shows the ranges for design parameters and objectives for output variables considered in the design parameter study. Each objective is set with the identical importance weight. Specifically, the first and third objectives about deformation and stress are adopted to enable that the EE works in the region below its elastic limit and at the same time enables cutting force measurement with satisfying stiffness, accuracy and linearity. Additionally, the second and fourth objectives related to the strain and the primary response frequency are considered to make sure that the system has superior performances such as high resolution and sensitivity, as well as satisfying dynamic performance.

**Table 2:** Design parameters and output variables.

	Design Parameters				Output Variables			
Parameter	Extended Circle Diameter	Diaphragm Thickness	Lamella Thickness	Lamella Length	Maximum Total Deformation (mm)	Minimum Elastic Strain (10^−6^ mm/mm)	Maximum Equivalent Stress (MPa)	The Primary Response Frequency (Hz)
Variable	P1	P2	P3	P4	P5	P6	P7	P8
Bound/Objective	40 < P1 < 50	1 < P2 < 4	1 < P3 < 3	7 < P4 < 12	P5 < 0.03	P6 > 100	P7 < 60	P8 > 500
Candidate 1	49.55	1.055	11.119	1.378	0.0273	159.884	39.572	744.3
Candidate 2	46.35	1.031	7.334	2.402	0.0265	134.272	54.719	1210.4
Candidate 3	47.95	1.149	10.297	2.658	0.0192	130.072	40.916	1043.7

**Figure 5:**
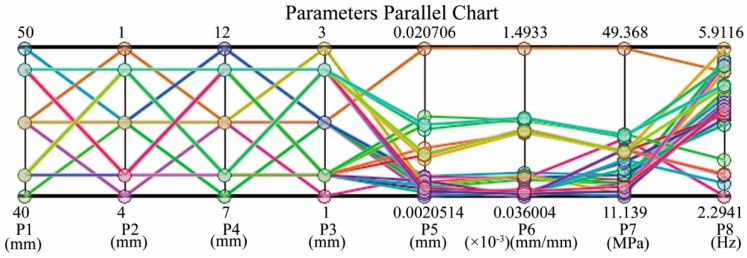
Parameters chart of SDO: design points with different colors generated via varied combination of the input parameters.

Figure 5 shows a graphical display of design parameters that demonstrate particular configurations of EE model associated with the corresponding output variables. Additionally, it also illustrates what each objective could achieve and if it entails sacrificing the objective of another output variable. Three best candidate sets with corresponding output variables that are most in alignment with all objectives are listed in the Table 2. Candidate set 1 is adopted in this design.

**Figure 6:**
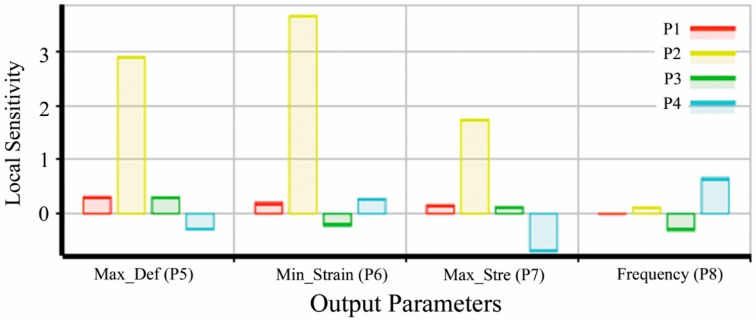
Sensitivity of the input parameters with respect to each output variables.

The influence ranks of input parameters over the output variables are calculated and shown in the Figure 6, and the greater positive sensitivity indicates greater impact on output variables positively, while more negative sensitivity means greater negative impact on output variables. From the sensitivity plot, it can be seen that the most noticeable parameter to each output variable are the membrane thickness as well as the lamella thickness.

**Figure 7:**
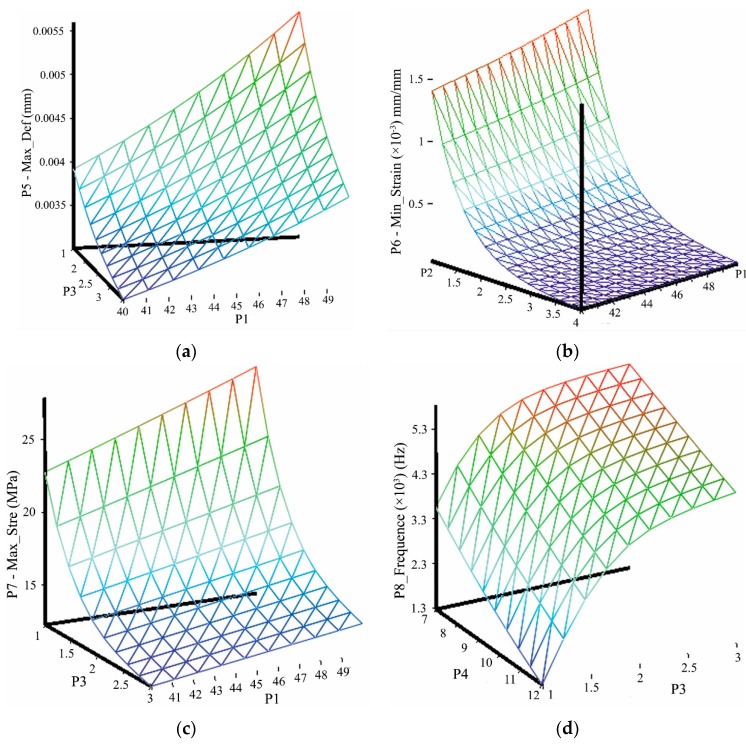
Relationships between design parameters and output variables: (**a**) Maximum deformation *versus* P1 and P3; (**b**) Minimum strain *versus* P1 and P2; (**c**) Maximum equivalent stress *versus* P1 and P3; (**d**) The first response frequency *versus* P4 and P5.

The relationships between design parameters and the variation of the output variables are illustrated in Figure 7. One can view the sensing system performances vary with respect to the design parameters intuitively, which is helpful to identify and understand specific changes to meet the corresponding requirements for the sensing system.

### 4.3. Dynamic Properties of the Sensor

The cutting force sensing system will respond dynamically when it is subject to applied cutting force in machining processes. Therefore, the identification of dynamic properties of the sensor is important both for dynamic measurement and control implementation thereafter.

A modal analysis is performed to measure and analyze the dynamic response of the sensor, e.g., vibration characteristics. A stiff support is set to the sensor base frame, and the interest responding frequencies is limited from the first to sixth frequencies, which are evaluated and displayed in Table 3. Moreover, the result also illustrate that the first two natural frequencies are almost the identical due to the symmetric structure of the sensor.

**Table 3:** The first six natural frequencies of the sensor.

Mode	1	2	3	4	5	6
Responding Frequency (Hz)	744.3	748.4	1291.8	1317.7	2869.9	2999.5

**Figure 8:**
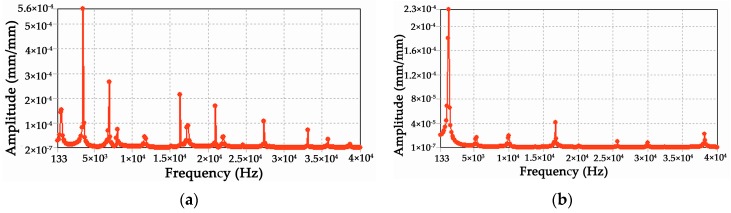
Harmonic response of the diaphragm under the measuring force component F*x* (**a**) and F*z* (**b**).

Furthermore, a harmonic analysis is performed with ANSYS to identify and predict the sustained dynamic behavior of the sensor subjected to harmonically varying loads. When a load and a fixed support are applied at the circular loop and base frame of the sensor(see Figure 4) respectively, the corresponding output normal elastic strain of the diaphragms are obtained (as illustrated in Figure 8) to verify whether the design can successfully withstand forced vibration and not undergo resonance.

## 5. Cutting Force Sensor Fabrication

### 5.1. The Arrangement Strategy of the Strain Gauges

**Figure 9:**
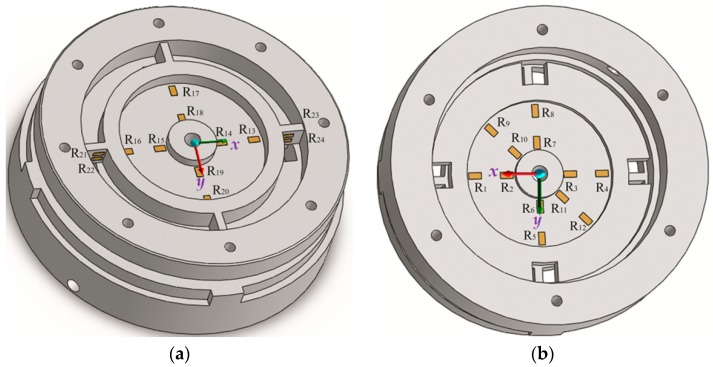
Arrangement strategy of the strain gauges: (**a**) Gauges emplacement on the upper diaphragm; (**b**) Gauges emplacement on the lower diaphragm.

Six groups of strain gauges are mounted in the areas of highest strain on the EE with considerations of maximizing the sensitivity to various components of cutting force and moment. 24 strain gauges (LY11, manufactured by HNM GmbH Inc., Balgheim, Germany) are bonded on the EE as shown in the Figure 9. Specifically, active strain gauges R1, R2, R3, and R4 (R5, R6, R7, and R8) are arranged on the lower diaphragm along the *x*-axis (*y*-axis) to detect force F*x* (force F*y*). Another four 45-degree active gauges R9, R10, R11, and R12 are bonded on the lower diaphragm along its radial axis to measure cutting force along *z*-axis F*z*. Also, four active strain gauges R13, R14, R15, and R16 (R17, R18, R19, and R20) are instrumented on the upper diaphragm along the *x*-axis (*y*-axis) to detect cutting moment about *y*-axis M*y* (cutting moment about *x*-axis M*x*). Additionally, four active gauges R21, R22, R23, and R24 are bonded on the lamella along its longitudinal direction to detect cutting moment about *z*-axis M*z*.

The bonded gauges resistances will vary when the external cutting forces are applied to the sensor. Table 4 summarizes the variations of the each gauge resistance due to six components of the cutting force with the FEA. In addition, the symbols “+” and “−” in the table indicate an increase and decrease of the gauge resistance, respectively. While the symbols “=” and “0” represent identical and no variation, respectively. It is revealed that the strains obtained from the derived equations are in agreement with the results from the FEA.

**Table 4:** Variations of resistances of strain gauges.

		F*x*	F*y*	F*z*	M*x*	M*y*	M*z*			F*x*	F*y*	F*z*	M*x*	M*y*	M*z*
F*x*-bridge	R_1_	+	0	+	0	0	+	M*x*-bridge	R_17_	0	+	+	+	0	+
	R_2_	-	0	-	0	0	+		R_18_	0	+	-	-	0	+
	R_3_	+	0	-	0	0	+		R_19_	0	-	-	+	0	+
	R_4_	-	0	+	0	0	+		R_20_	0	-	+	-	0	+
F*y*-bridge	R_5_	0	+	+	0	0	+	M*y*-bridge	R_13_	-	0	+	0	+	+
	R_6_	0	-	-	0	0	+		R_14_	-	0	-	0	-	+
	R_7_	0	+	-	0	0	+		R_15_	+	0	-	0	+	+
	R_8_	0	-	+	0	0	+		R_16_	+	0	+	0	-	+
F*z*-bridge	R_9_	+	+	+	0	0	+	M*z*-bridge	R_21_	=	=	+	0	+	-
	R_10_	-	-	-	0	0	+		R_22_	=	=	+	0	+	-
	R_11_	+	+	-	0	0	+		R_23_	=	=	+	0	+	+
	R_12_	-	-	+	0	0	+		R_24_	=	=	+	0	+	+

### 5.2. The Measuring Circuit

**Figure 10:**
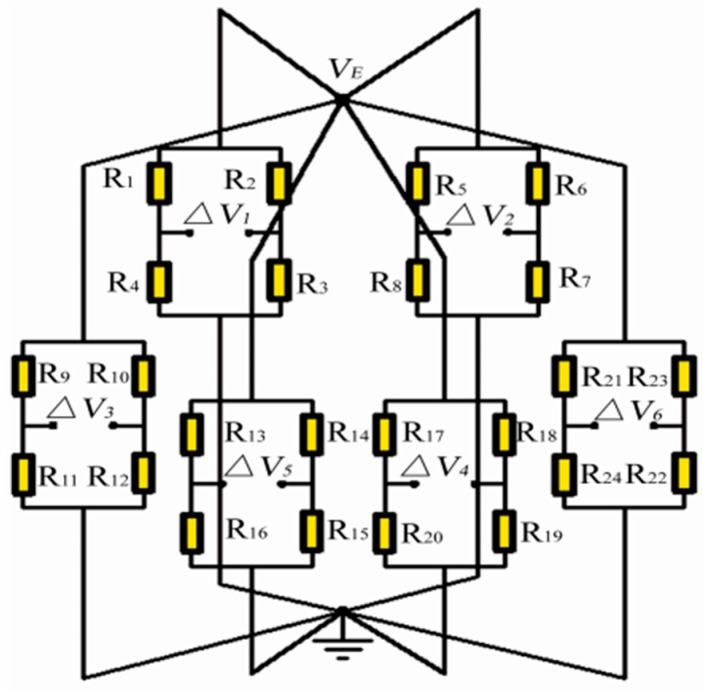
Electrical circuits of the strain gauges.

In practice, strain gauges measurements always involve quantities smaller than a few milli-strain (10^−3^ mm/mm), and their corresponding changes in resistance are extremely minute. Therefore, strain gauges are electrically connected to form six full-bridge configuration Wheatstone bridge circuits to detect six components of cutting load with high accuracy independently, as shown in Figure 10. The excitation diagonals of the Wheatstone bridges are connected to a voltage excitation, and the corresponding outputs voltage ΔV*i* will appear on the measurement diagonals of the bridges.

#### 5.2.1. Subjected to a Cutting Force F*x*

While the system is individually applied with the tangential cutting force F_x_ (similar to F_y_), the resistances changes in the way as defined in the Table 4. Therefore, the output signals can be expressed as follows:

(21) $$\Delta V_{1}=\frac{V_{E}}{4}\left(\frac{\Delta R_{1}}{R_{1}}-\frac{\Delta R_{2}}{R_{2}}+\frac{\Delta R_{3}}{R_{3}}-\frac{\Delta R_{4}}{R_{4}}\right)=\frac{V_{E}}{4}\left[2{\left(\frac{\Delta R_{1}}{R_{1}}\right)}_{\varepsilon }-2{\left(\frac{\Delta R_{2}}{R_{2}}\right)}_{\varepsilon }\right]=\frac{V_{E}}{4}\left[2\left|{\left(\frac{\Delta R_{1}}{R_{1}}\right)}_{\varepsilon }\right|+2\left|{\left(\frac{\Delta R_{2}}{R_{2}}\right)}_{\varepsilon }\right|\right]$$

(22) $$\Delta V_{2}=\frac{V_{E}}{4}\left(\frac{\Delta R_{5}}{R_{5}}-\frac{\Delta R_{6}}{R_{6}}+\frac{\Delta R_{7}}{R_{7}}-\frac{\Delta R_{8}}{R_{8}}\right)=0$$

(23) $$\Delta V_{3}=\frac{V_{E}}{4}\left(\frac{\Delta R_{9}}{R_{9}}-\frac{\Delta R_{10}}{R_{10}}+\frac{\Delta R_{11}}{R_{11}}-\frac{\Delta R_{12}}{R_{12}}\right)=0$$

(24) $$\Delta V_{4}=\frac{V_{E}}{4}\left(\frac{\Delta R_{17}}{R_{17}}-\frac{\Delta R_{18}}{R_{18}}+\frac{\Delta R_{19}}{R_{19}}-\frac{\Delta R_{20}}{R_{20}}\right)=0$$

(25) $$\Delta V_{5}=\frac{V_{E}}{4}\left(\frac{\Delta R_{13}}{R_{13}}-\frac{\Delta R_{14}}{R_{14}}+\frac{\Delta R_{15}}{R_{15}}-\frac{\Delta R_{16}}{R_{16}}\right)=0$$

(26) $$\Delta V_{6}=\frac{V_{E}}{4}\left(\frac{\Delta R_{21}}{R_{21}}-\frac{\Delta R_{22}}{R_{22}}+\frac{\Delta R_{23}}{R_{23}}-\frac{\Delta R_{24}}{R_{24}}\right)=0$$

where the ${\left(\Delta R_{i}/R_{i}\right)}_{\varepsilon }$ represents the resistance variation rate of the gauge R_i_, caused by the strain change. The above analysis shows that only the F*x*-bridge is dependent on the applied cutting force F*_x_*, and the outputs of else bridge circuits are independent.

#### 5.2.2. Under the Normal Cutting Force F*z*

Under a cutting force F*z* action the corresponding output voltages of the sensor can be written as follows:

(27) $$\Delta V_{3}=\frac{V_{E}}{4}\left(\frac{\Delta R_{9}}{R_{9}}-\frac{\Delta R_{10}}{R_{10}}+\frac{\Delta R_{11}}{R_{11}}-\frac{\Delta R_{12}}{R_{12}}\right)=\frac{V_{E}}{4}\left[2{\left(\frac{\Delta R_{9}}{R_{9}}\right)}_{\varepsilon }-2{\left(\frac{\Delta R_{10}}{R_{10}}\right)}_{\varepsilon }\right]=\frac{V_{E}}{4}\left[2\left|{\left(\frac{\Delta R_{9}}{R_{9}}\right)}_{\varepsilon }\right|+2\left|{\left(\frac{\Delta R_{10}}{R_{10}}\right)}_{\varepsilon }\right|\right]$$

(28) $$\Delta V_{1}=\Delta V_{2}=\Delta V_{4}=\Delta V_{5}=\Delta V_{6}=0$$

#### 5.2.3. Under the Cutting Moment about the Tangential Axis M*x* (Similar to M*y*)

Under the cutting moment M*x* action the sensor output signals can be derived as:

(29) $$\Delta V_{4}=\frac{V_{E}}{4}\left(\frac{\Delta R_{17}}{R_{17}}-\frac{\Delta R_{18}}{R_{18}}+\frac{\Delta R_{19}}{R_{19}}-\frac{\Delta R_{20}}{R_{20}}\right)=\frac{V_{E}}{4}\left[2{\left(\frac{\Delta R_{17}}{R_{17}}\right)}_{\varepsilon }-2{\left(\frac{\Delta R_{18}}{R_{18}}\right)}_{\varepsilon }\right]=\frac{V_{E}}{4}\left[2\left|{\left(\frac{\Delta R_{17}}{R_{17}}\right)}_{\varepsilon }\right|+2\left|{\left(\frac{\Delta R_{18}}{R_{18}}\right)}_{\varepsilon }\right|\right]$$

(30) $$\Delta V_{1}=\Delta V_{2}=\Delta V_{3}=\Delta V_{5}=\Delta V_{6}=0$$

#### 5.2.4. Subjected to a Cutting Moment M*z*

Under a cutting moment an M*z* action the lamellas with strain gauges will be subjected to a tensile/compressive stress. As a result, the output signals of the sensor can be expressed as:

(31) $$\Delta V_{6}=\frac{V_{E}}{4}\left(\frac{\Delta R_{21}}{R_{21}}-\frac{\Delta R_{23}}{R_{23}}+\frac{\Delta R_{22}}{R_{22}}-\frac{\Delta R_{24}}{R_{24}}\right)=\frac{V_{E}}{4}\left[2{\left(\frac{\Delta R_{21}}{R_{21}}\right)}_{\varepsilon }-2{\left(\frac{\Delta R_{23}}{R_{23}}\right)}_{\varepsilon }\right]=\frac{V_{E}}{4}\left[2\left|{\left(\frac{\Delta R_{21}}{R_{21}}\right)}_{\varepsilon }\right|+2\left|{\left(\frac{\Delta R_{23}}{R_{23}}\right)}_{\varepsilon }\right|\right]$$

(32) $$\Delta V_{1}=\Delta V_{2}=\Delta V_{3}=\Delta V_{4}=\Delta V_{5}=0$$

Hence, each force/moment component of the cutting load can only generate an output voltage of the corresponding bridge circuit, which means the coupling among the sensor components is negligible in theory. In addition, it is seen that the electrical circuits characterized with intrinsic compensation for temperature drift.

## 6. Calibration and Characterization of the Sensor

**Figure 11:**
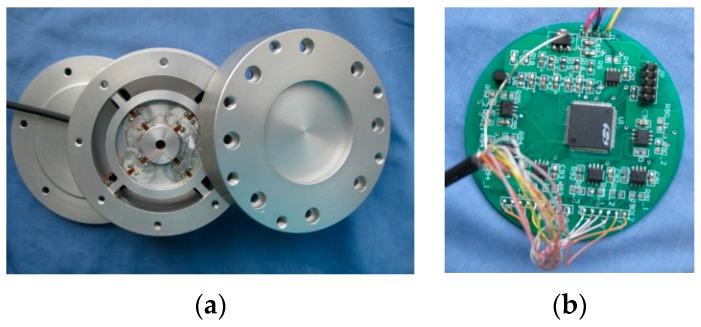
Prototype of the sensor system for cutting force measurement: (**a**) the upper adapter, EE and the lower adapters; (**b**) the integrated electric circuit.

A prototype of the sensor system for cutting force measurement in machining processes with an integrated electric circuit has been constructed, as shown in Figure 11. The sensor has a total size of Φ 80 mm × 42 mm. The measurement range of the system is set to 0–250 N for cutting force F*z*, ±200 N for cutting forces in shear directions (F*x* and F*y*), and ±10 Nm for cutting moments around normal axis, and ±8 Nm for cutting moments around the shear axis.

**Figure 12:**
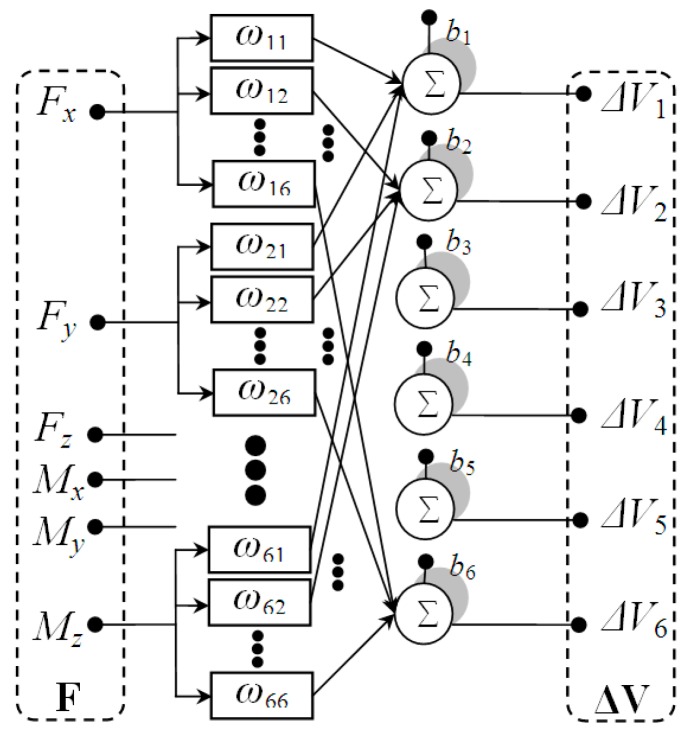
Coupling model of multi-dimensional cutting force sensor system.

Precise calibration and decoupling of multi-dimensional cutting force sensor system is critical and must be addressed. The coupling effects among cutting force and moment components are depicted in Figure 12. The relationship between the applied cutting load and output of the system could be expressed as:

(33) ΔV=FW+b

With the method and procedure previously proposed in [17], calibration and decoupling experiment of the proposed system are carried out. Table 5 shows the outputs of the sensor under different applied cutting forces and moments. Specifically, the outputs of the components have a good symmetry about the zero point.

**Table 5:** The outputs of the sensor.

Component	F*x*	F*y*	F*z*	M*x*	M*y*	M*z*
Applied load	-	-	-	-	-	−10 Nm
Corresponding output	-	-	-	-	-	−3.40 V
Applied load	−200 N	−200 N	0 N	−8 Nm	−8 Nm	−8 Nm
Corresponding output	−4.48 V	−4.35 V	−0.0034 V	−4.03 V	−3.91 V	−2.72 V
Applied load	−150 N	−150 N	50 N	−6 Nm	−6 Nm	−6 Nm
Corresponding output	−3.41 V	−3.31 V	0.73 V	−3.08 V	−2.98 V	−2.04 V
Applied load	−100 N	−100 N	100 N	−4 Nm	−4 Nm	−4 Nm
Corresponding output	−2.35 V	−2.28 V	1.40 V	−2.12 V	−2.06 V	−1.36 V
Applied load	−50 N	−50 N	150 N	−2 Nm	−2 Nm	−2 Nm
Corresponding output	−1.27 V	−1.25 V	2.07 V	−1.15 V	−1.10 V	−0.68 V
Applied load	0 N	0 N	200 N	0 Nm	0 Nm	0 Nm
Corresponding output	−0.00153 V	−0.00168 V	2.72 V	0.0006 V	0.00229 V	0.0032 V
Applied load	50 N	50 N	250 N	2 Nm	2 Nm	2 Nm
Corresponding output	1.28 V	1.26 V	3.35 V	1.15 V	1.11 V	0.66 V
Applied load	100 N	100 N	-	4 Nm	4 Nm	4 Nm
Corresponding output	2.40 V	2.28 V	-	2.11 V	2.05 V	1.37 V
Applied load	150 N	150 N	-	6 Nm	6 Nm	6 Nm
Corresponding output	3.43 V	3.32 V	-	3.09 V	2.99 V	2.08 V
Applied load	200 N	200 N	-	8 Nm	8 Nm	8 Nm
Corresponding output	4.5 V	4.37 V	-	4.03 V	3.91 V	2.80 V
Applied load	-	-	-	-	-	10 Nm
Corresponding output	-	-	-	-	-	3.50 V

Table 6 shows the performance of the proposed cutting force measurement system. From the table, these values show that the proposed system is superior in the maximum coupling and nonlinearity errors. Besides, the system still suffers from slight coupling due to the monolithic EE structure and the attachment error of the strain gauges.

**Table 6:** The performance of the system.

Component	F*x*	F*y*	F*z*	M*x*	M*y*	M*z*
Sensitivity	0.02245 V·N^−1^	0.02180 V·N^−1^	0.0134 V·N^−1^	0.5038 V·(Nm)^−1^	0.4888 V·(Nm)^−1^	0.345 V·(Nm)^−1^
Maximum coupling error	1.07%	1.38%	0.41%	1.47%	1.09%	0.39%
Maximum nonlinearity error	1.75%	1.94%	1.87%	−1.77%	−1.69%	1.15%

## 7. Conclusions

In the present study, a new sensor system for cutting force measurement with a novel decoupling EE has been proposed. The sensor is intended to be used in machining processes for measuring tangential and normal forces F*x*, F*y* and F*z* as well as the moments terms M*x*, M*y* and M*z* simultaneously. By performing a Simulation-Driven Optimization in conjunction with the FEA, the best combination of design parameters is efficiently and scientifically determined. It is revealed that the proposed EE structure with double circular diaphragms may be adopted by other force/moment sensors. As shown in the Table 7, the obtained results demonstrate that the proposed system has satisfied performance and can be utilized to detect static cutting F/M.

**Table 7:** The performance comparisons with some proven reference sensors.

Developer	Approach & Measurement Principle	Size (mm)	No. of Axes	Sensitivity	Maximum Relative Error
Tuysuz, Altintas, Feng [9]	Indirect & prediction model	n.a.	5	n.a.	8.5%
Rao, Gao, Friedrich [13]	Direct & Piezoelectric	Integrated into system	1	7 mV/gm	9.8%
Kim, Kim [15]	Direct & strain gauge and piezo-film accelerometer	40 × 70 × 26	2	3 mV·N^−1^	n.a.
Yaldız, Ünsaçar [23]	Direct & strain gauge and piezoelectric accelerometer	100 × 100 × 50	3	0.1 mV·N^−1^	<5%
Liu, Zhou, Tao, Tan [24]	Direct & strain gauge and fiber Bragg grating sensor	n.a.	3	n.a.	6.23%
Our approach	Direct & strain gauge	Φ 80 × 42	6	0.0134 V·N^−1^ and 0.345 V (Nm)^−1^	<5%

The sensor system is now being applied to measure cutting loads in machining processes, and reliable results are expected based on the findings reported in this manuscript. For more information about the dynamic cutting F/M measurement and actual cutting experiments, readers should look to our future work with this sensor system.
